# Supplementary material for: A meta-learning approach for genomic survival analysis
Source: Nat Commun. 2020 Dec 11;11:6350. doi: 10.1038/s41467-020-20167-3 (PMC7733508; doi:10.1038/s41467-020-20167-3)
Supplement: Supplementary file 1 — Supplementary Information [file 41467_2020_20167_MOESM1_ESM.pdf]

A meta-learning approach for genomic survival analysis

Supplementary Figure

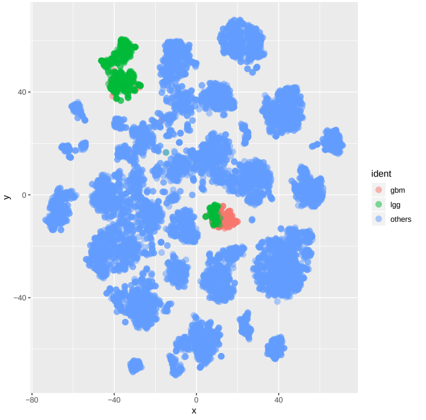

(a) LGG/GBM

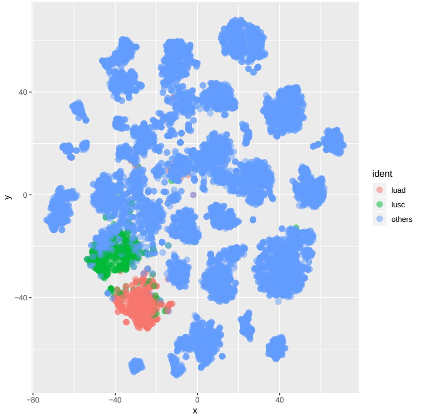

(b) LUAD/LUSC

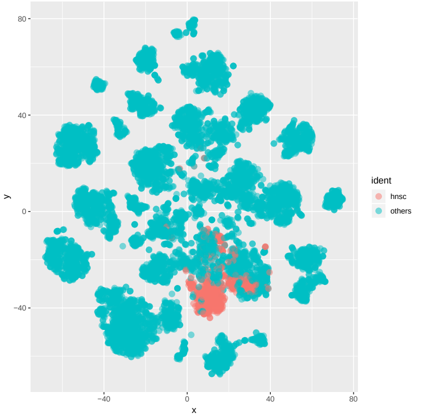

(c) HNSC

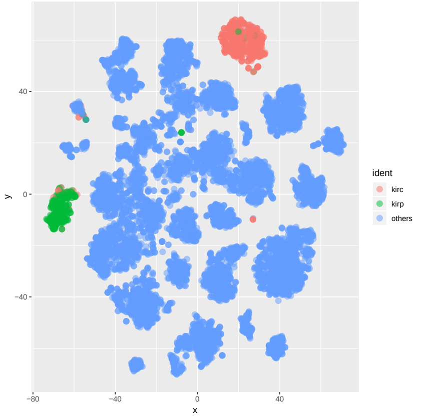

(d) KIRC/KIRP

Supplementary Fig. 1: Mapping of 33 types of cancers' gene expression data with a t-distributed stochastic neighbor embedding (t-SNE), highlighting (1a) LGG/GBM versus the rest of cancers; (1b) LUAD/LUSC versus the rest of cancers; (1c) HNSC versus the rest of cancers; (1d) KIRC/KIRP versus the rest of cancers. Renal cell carcinomas (i.e. KIRC and KIRP) are farther apart from other types of cancers, and also more heterogeneous within, thus information transfer from other cancers is considered more difficult.

Supplementary Tables

Supplementary Table 1: Glioma gene set over-representation (p <= 0.01). P values are calculated by the one-sided Fisher's exact test with the hypergeometric distribution. P value (adjusted) are adjusted for multiple comparisons.

**Pathway**	**Gene set size**	**p value**	**p value (adjusted)**	**Jaccard Index**	**Overlapping**	**Number of overlapping**	**Genes**	**Source**
NOSTRIN mediated eNOS trafficking	6	0.008571329	1	0.001744186	NOSTRIN; WASL; CAV1	3	lowriskgenes	REACTOME
Disinhibition of SNARE formation	5	0.008571329	1	0.001744186	STXBP3; PRKCB; STX4	3	lowriskgenes	REACTOME
RUNX1 regulates transcription of genes involved in differentiation of myeloid cells	6	0.008571329	1	0.001744186	PRKCB; CBFB; CREBBP	3	lowriskgenes	REACTOME
HSP90 chaperone cycle for steroid hormone receptors (SHR)	19	0.008593	1	0.003466205	NR3C2; NR3C1; HSPA1A; HSP90AA1; HSPA1B; FKBP4	6	lowriskgenes	REACTOME
Dectin-2 family	29	0.008593	1	0.003466205	CLEC10A; MUC20; MUC5B; FCER1G; MUC4; SYK	6	lowriskgenes	REACTOME
PAOs oxidise polyamines to amines	2	0.010013404	1	0.001164144	PAOX; SMOX	2	lowriskgenes	REACTOME
TWIK-releated acid-sensitive K+ channel (TASK)	2	0.010013404	1	0.001164144	KCNK9; KCNK3	2	lowriskgenes	REACTOME
Viral carcinogenesis - Homo sapiens (human)	201	0.00190752	0.612313978	0.017094017	IL6ST; IKBKG; GSN; DLG1; STAT3; PIK3CB; PIK3CD; ATP6V0D2; CREB1; KRAS; HLA-C; GTF2E2; EGR3; CCNE1; HIST1H2BL; HIST1H2BH; TBPL1; TBP; CDC20; HDAC5; HDAC6; CDKN1B; C3; RANBP1; MRPS18B; KAT2B; EP300; BAD; HIST2H2BE; YWHAB; TRAF3; ATF6B	32	highriskgenes	KEGG
Herpes simplex infection - Homo sapiens (human)	185	0.007380866	1	0.013579576	IKBKG; EP300; OAS2; CSNK2A1; IFNAR2; SOCS3; RNASEL; IFIT1; GTF2IRD1; TNFRSF1A; TBP; TAF10; MAPK10; STAT2; SKP1; OAS3; HLA-C; C3; TBPL1; CYCS; IL15; HLA-DOA; TRAF3; POU2F3; CFP	25	highriskgenes	KEGG
Cytokine Signaling in Immune system	458	0.00015883	0.299552844	0.031476998	IL6ST; IFNAR2; PIK3CB; PIK3CD; IRAK2; IRAK4; CAMK2A; SNAP25; TNFSF13; TNFSF15; IFIT1; HCK; SIGIRR; FLNB; PELI2; CISH; BRWD1; IL2RB; HLA-C; MAP3K14; IFITM3; IFITM1; CREB1; TNFRSF25; UBA52; IL10RB; TRIM8; MEF2A; SKP1; TNFSF9; TNFRSF6B; TNFSF4; HIST1H3H; OSM; IL27; MAP3K8; MAP3K3; TRIM29; INPP5D; IKBKG; TNFRSF18; GBP2; GBP7; PTPN2; PTPN6; STAT3; STAT2; TNFRSF1A; IL15; IL1RAP; CASP1; S100A12; OAS3; OAS2; RNASEL; SOCS3; EIF4G1; IL20RB; UBE2L6; TRIM46; MAPK14; MAPK10; PSMB8; ADAM17; TRAF3	65	highriskgenes	REACTOME
Signaling by NOTCH	119	0.001166907	1	0.012195122	MOV10; FURIN; DLGAP5; MYC; CREB1; HDAC5; UBA52; YBX1; PRKCI; ARRB2; KAT2B; NCSTN; HDAC6; TLE1; NCOR2; POFUT1; SKP1; LFNG; EP300; ADAM17; ST3GAL4; PBX1	22	highriskgenes	REACTOME
Regulation of mRNA stability by proteins that bind AU-rich elements	37	0.002709399	1	0.005737235	TNFSF13; PABPC1; MAPK14; ANP32A; EXOSC3; EXOSC7; PARN; YWHAB; DCP2; EIF4G1	10	highriskgenes	REACTOME
Interferon alpha/beta signaling	70	0.002974208	1	0.007390563	HLA-C; IFNAR2; IFIT1; STAT2; OAS3; OAS2; IFITM3; IFITM1; PTPN6; SOCS3; RNASEL; PSMB8; GBP2	13	highriskgenes	REACTOME

Supplementary Table 1: Glioma gene set over-representation (p <= 0.01). P values are calculated by the one-sided Fisher's exact test with the hypergeometric distribution. P value (adjusted) are adjusted for multiple comparisons. (continued)

**Pathway**	**Gene set size**	**p value**	**p value (adjusted)**	**Jaccard Index**	**Overlapping**	**Number of overlapping**	**Genes**	**Source**
Immune System	1827	0.003342285	1	0.059848734	AGL; UBE2Q2; CD226; PIK3CB; PIK3CD; MUC1; ACLY; FBXL12; FBXL19; PSMD12; SIGIRR; PELI2; ACTR10; CD93; CFP; ASB3; CNPY3; IL10RB; TRIM8; MEF2A; SH3RF1; PRDX6; WIPF3; WIPF1; EP300; ATP6V0D2; CUL2; TRIM29; ARHGAP9; LAIR2; C4BPA; PTPN2; PTPN6; PTPN4; C3; FBXO41; SLPI; FCGR3B; MAPKAP1; IL1RAP; SEC61A1; UBR2; KRAS; SOCS3; ARPC1A; EIF4G1; IL20RB; ARPC2; RNF123; MAPK14; MAPK10; DAPP1; P2RX7; TRAF3; IL6ST; COMMD9; CAMK2A; CISH; PSMC3; SEC61G; MAP3K14; IKBKG; TMEM173; GNLY; VAT1; UBA52; NCSTN; SKP1; LCN2; HIST1H3H; MAP3K8; MAP3K3; INPP5D; CARD9; PSMB8; UBA6; UBE2C; IL15; ATP11A; RNASEL; FBXL3; TLR1; FAF2; CDC20; DTX3L; ADAM17; MUC16; RASGRP3; TANK; HCK; GZMM; SPSB2; BRWD1; C5AR1; CDH1; IFITM3; IFITM1; RNF19A; UNC93B1; VNN1; UBE2E3; TNFRSF6B; YWHAB; AIM2; VAMP8; ABI1; IL27; PECAM1; PLD1; RNF217; RNF213; DOCK2; SMURF1; FTH1; PPIA; WASF2; CASP4; CASP1; HECTD3; S100A12; OAS3; OAS2; CAPN1; PI3; RNF220; TRIM46; LRMP; HLA-C; UBE2G1; IFNAR2; IRAK2; OSM; IRAK4; IL2RB; SNAP25; TNFSF13; TNFSF15; IFIT1; NFASC; FLNB; GGH; TNFAIP6; PSMD2; ALDH3B1; NOS3; CREB1; PRCP; TNFRSF25; C1QA; ZBTB16; TNFSF9; TNFSF4; TRIM69; RNF34; XRCC5; CLEC4G; CLEC4A; TNFRSF18; GBP2; GBP7; HERC1; HERC6; CR2; TNFRSF1A; STAT3; STAT2; CD68; HLA-DOA; CHIT1; LAT2; GSN; GOLGA7; APEH; UBE2L6; PTPRN2; FCGR2B; CD58; DOK3; SLC11A1; SIGLEC9; SIGLEC7; FOLR3	182	highriskgenes	REACTOME
TNFs bind their physiological receptors	29	0.004906229	1	0.004608295	TNFSF13; TNFRSF25; TNFSF15; TNFRSF18; TNFSF9; TNFRSF6B; TNFSF4; TNFRSF1A	8	highriskgenes	REACTOME
Voltage gated Potassium channels	44	0.005076968	1	0.005169443	KCNG2; KCNA2; KCNS1; KCND2; KCNQ3; KCNQ1; KCNF1; KCNH7; KCNH2	9	highriskgenes	REACTOME
Collagen degradation	35	0.006191992	1	0.004605642	MMP15; MMP10; ADAM17; FURIN; COL18A1; COL9A2; COL16A1; COL17A1	8	highriskgenes	REACTOME
TNFR2 non-canonical NF-kB pathway	50	0.007903028	1	0.00627138	TNFSF13; TNFRSF25; TNFSF15; TNFRSF18; SKP1; TNFSF9; TNFSF4; TRAF3; TNFRSF1A; TNFRSF6B; MAP3K14	11	highriskgenes	REACTOME
HATs acetylate histones	142	0.00981636	1	0.011043622	DR1; YEATS2; YEATS4; KAT2B; EP400; TADA2A; HIST1H2BL; HIST1H2BH; PHF20; TAF10; ATXN7L3; EP300; HIST2H2AA3; HIST2H2BE; HIST1H2AL; HIST1H2AG; HIST1H2AC; HIST1H3H; MEAF6; RUVBL2	20	highriskgenes	REACTOME
Mammary gland development pathway - Involution (Stage 4 of 4)	10	0.001626496	0.977524312	0.002905288	STAT3; SOCS3; IL6ST; CDH1; MYC	5	highriskgenes	Wikipathways
Integrated Breast Cancer Pathway	66	0.006464185	1	0.007369615	RASGRP3; NUP85; XRCC3; ODC1; EP300; RAD54L; GADD45A; FOSL1; CDH1; MYC; CREB1; KRAS; AHR	13	highriskgenes	Wikipathways

Supplementary Table 2: Glioma gene set enrichment analysis (GSEA) (p <= 0.01). P values are calculated by the permutation test (nperm=100000). P value (adjusted) are adjusted for multiple comparisons.

Pathway	p value	p value (adjusted)	ES	NES	n More Extreme	Size	Leading Edge Genes	Source
Renin-angiotensin system - Homo sapiens (human)	0.010151278	0.74865674	0.55331802	1.713839711	511	19	PRCP; KLK1; THOP1; PREP; ANPEP; CTSA; ENPEP; CPA3; CTSG; AGT; NLN	KEGG
Viral carcinogenesis - Homo sapiens (human)	0.001408837	0.415606778	0.309546872	1.528384025	71	188	CCNE1; HIST1H2BL; GTF2E2; HDAC6; YWHAB; KRAS; TBP; CDC20; C3; HIST1H2BH; TRAF3; RANBP1; EP300; CDKN1B; STAT3; IL6ST; HIST2H2BE; ATP6V0D2; DLG1; TBPL1; BAD; PIK3CB; HDAC5; GSN; IKBKG; HLA-C; CREB1; KAT2B; PIK3CD; ATF6B; EGR3; MRPS18B; HLA-B; SKP2; HLA-A; EIF2AK2; CDKN2B; TP53; RHOA; PRKACA; POLB; TRAF1; CDK6; BAX; TRADD; MAPKAPK2; SNW1; PMAIP1; GRB2; ACTN4; JAK3; CREB3L2; TRAF2; NRAS; PIK3R1; GTF2E1; NFKBIA; HDAC7; GTF2H3; IRF9; PRKACB; YWHAZ; NFKB1; DDB1; SCRIB; BAK1	KEGG
Hepatitis C - Homo sapiens (human)	0.004599636	0.452297534	0.321690611	1.513911872	234	134	YWHAB; KRAS; IFNAR2; TRAF3; BID; RNASEL; CYCS; OAS2; TNFRSF1A; STAT3; OAS3; SOCS3; STAT2; BAD; PIK3CB; MYC; IFIT1; IKBKG; PIK3CD; CLDN11; EIF2AK1; EIF2AK2; FAS; PPP2R2B; EIF2AK3; TP53; PPP2R1A; CDK6; BAX; TRADD; PSME3; GRB2; CLDN15; E2F3; TRAF2; CLDN16; NRAS; PIK3R1; TRAF6; CXCL10; NFKBIA; NR1H3; IRF9; YWHAZ; NFKB1	KEGG
Epstein-Barr virus infection - Homo sapiens (human)	0.003443486	0.452297534	0.302236718	1.4859104	175	182	CCNE1; HLA-DOA; CD58; IFNAR2; TRAF3; CR2; MAPK10; BID; CYCS; OAS2; CDKN1B; PSMD2; STAT3; OAS3; NCOR2; MAP3K14; PSMC3; STAT2; PIK3CB; MYC; IKBKG; PSMD12; HLA-C; GADD45A; IRAK4; PIK3CD; MAPK14; PSMD8; HLA-B; CALR; SKP2; NFKBIE; PSMD4; SIN3A; HLA-A; DDB2; EIF2AK2; PLCG2; PSMD3; FAS; TP53; ENTPD8; CDK6; BAX; TRADD; PSMC4; SNW1; NFKBIB; ITGAL; JAK3; E2F3; TRAF2; PIK3R1; TRAF6; CXCL10; NFKBIA; BTK; HLA-DRB1; SAP30; IRF9	KEGG
Neutrophil degranulation	0.007895092	0.836411609	0.239588011	1.304953275	409	448	FTH1; PLD1; PRDX6; COMMD9; LCN2; VAT1; ATP11A; S100A12; LRMP; ALDH3B1; CFP; TMEM173; AGL; PRCP; GGH; C3; CD58; SLPI; DOCK2; GOLGA7; CHIT1; XRCC5; APEH; ACLY; FOLR3; SNAP25; CAPN1; VNN1; PPIA; PSMD2; SLC11A1; NFASC; NCSTN; PSMC3; PECAM1; CD68; DOK3; SIGLEC9; GSN; ARHGAP9; PSMD12; VAMP8; HLA-C; C5AR1; PTPN6; CD93; ACTR10; FAF2; TNFAIP6; MAPK14; FCGR3B; PTPRN2; HLA-B; CYFIP1; RHOG; GALNS; TMBIM1; VCL; CAP1; HLA-A; OSTF1; CCT2; HPSE; ANPEP; COTL1; PLAU; CTSA; PYGB; PLEKHO2; RAB10; C1orf35; CD36; SIGLEC5; CTSZ; AGA; RAP2C; ANXA2; RAP1A; PSMD3; XRCC6; TXNDC5; ITGAV; FCGR2C; RHOA; DNAJC5; BPI; ENPP4; RAB27A; FUCA2; ANO6; ATP11B; GRN; ITGAM; CD300A; CYB5R3; LILRB3; RNASET2; HK3; CLEC4D; ATP8B4; MMP9; ALDOA; SERPINB1; NPC2; NIT2; CDA; LILRA3; ITGAL; TBC1D10C; SERPINA3; S100A9; HRNR; SIRPA; PAFAH1B2; MLEC; HGSNAT; OLR1; NRAS; CYBA; CTSG; IQGAP2; RAB3A; LAMP1; CLEC5A; ALOX5; LTA4H; SLC2A5; SERPINA1; PA2G4; LAMP2; NFKB1; DSN1; PSMA2; RAB6A; SYNGR1; PGAM1; FPR1; TMEM179B; S100A11; EPX; ARL8A; NME1-NME2; ATG7; SLCO4C1; RNASE3; FABP5; CD59; DSP; CCT8; RAB37	Reactome

Supplementary Table 2: Glioma gene set enrichment analysis (GSEA) (p <= 0.01). P values are calculated by the permutation test (nperm=100000). P value (adjusted) are adjusted for multiple comparisons. (continued)

Pathway	p value	p value (adjusted)	ES	NES	n More Extreme	Size	Leading Edge Genes	Source
Cytokine Signaling in Immune system	0.00922317	0.836411609	0.241344672	1.304909082	477	414	IL15; MEF2A; PELI2; TRIM29; S100A12; OSM; IL2RB; CAMK2A; HCK; IFNAR2; TRAF3; GBP2; GBP7; MAPK10; UBE2L6; EIF4G1; PTPN2; RNASEL; FLNB; OAS2; TNFSF4; TNFRSF1A; SNAP25; TNFRSF18; INPP5D; IL20RB; CISH; TNFRSF6B; TNFRSF25; STAT3; OAS3; TNFSF9; IRAK2; MAP3K14; IL6ST; BRWD1; SOCS3; STAT2; ADAM17; PIK3CB; TRIM8; TNFSF13; IFIT1; IKBKG; PSMB8; SIGIRR; SKP1; IL27; MAP3K8; HIST1H3H; HLA-C; TRIM46; IL10RB; CREB1; IRAK4; PIK3CD; PTPN6; MAPK14; MAP3K3; CASP1; IFITM3; UBA52; IFITM1; IL1RAP; TNFSF15; CD27; HLA-B; SAA1; EGR1; IL18; UBE2E1; HIST1H3J; HERC5; IRF1; ARIH1; HLA-A; EIF2AK2; IL18BP; IL1A; IFI30; PELI1; GBP6; FYN; TNFRSF13C; PPP2R1A; PRKACA; HIST2H3D; IL18R1; LIF; IL10; SDC1; HIST1H3I; CSF3; EIF4A2; MAPKAPK2; NCAM1; RIPK2; TNFSF8; EIF4G3; NFKBIB; GRB2; IL2RG; IL12RB2; TAB3; JAK3; VRK3; UBA7; USP18; TRAF2; SUMO1; CNTFR; PIK3R1; TRAF6; NFKBIA; GAB2; IL1RN; HLA-DRB1; IRF9; TRIM38; YWHAZ; CD4; NFKB1; TRIM5; CLCF1; HAVCR2; TMEM189	Reactome
Collagen degradation	0.005869449	0.836411609	0.499213775	1.730725335	296	29	FURIN; COL18A1; COL16A1; ADAM17; MMP15; COL9A2; COL17A1; MMP10; TMPRSS6; MMP12	Reactome
Endogenous sterols	0.009686916	0.836411609	0.532156676	1.717629863	486	22	AHR; CYP46A1; CYP7B1; CYP11A1; CYP21A2; FDXR; CYP27A1; PTGIS	Reactome
RHO GTPases Activate WASPs and WAVEs	0.004305492	0.836411609	0.477078151	1.73230666	217	35	ABI1; ARPC1A; WIPF3; WASF2; WIPF1; ARPC2; CYFIP1; ACTR3; PTK2; ACTB; ACTG1; GRB2; WASF1; BTK	Reactome
Metabolism of steroid hormones	0.001403689	0.836411609	0.569680106	1.903149269	70	25	HSD11B2; STAR; STARD3NL; CYP11A1; CYP21A2; HSD17B2; HSD11B1; FDXR	Reactome
G1/S Transition	0.008919762	0.836411609	0.370382713	1.558707807	451	70	CCNE1; POLE2; CDKN1B; RRM2; CDC6; FBXO5; MYC; MCM5; WEE1; SKP1; UBA52; SKP2; DHFR; TFDP2; MAX; PRIM2; CCNB1; PPP2R1A	Reactome
Transcriptional Regulation by TP53	0.008834161	0.836411609	0.254178091	1.341368606	456	325	CCNE1; POLR2G; PRKAA1; RABGGTB; CDK12; MEAF6; RNF34; YWHAB; TBP; PRDM1; PPP1R13L; DNA2; PHF20; E2F7; PRDX1; BID; TAF7; EP300; CYCS; TAF10; MOV10; EHMT2; CASP6; CDKN1B; TP73; TNFRSF10B; MAPKAP1; CHEK2; RRAGD; PIP4K2A; DDIT4; TNFRSF10A; CSNK2A1; GADD45A; MAPK14; BANP; CASP1; BRD7; TP53I3; UBA52; CCNK; PLK2; FANCC; PTEN; CDK9; TNFRSF10D; DDB2; TP53BP2; IGFBP3; BRPF3; AURKA; TAF6; BRPF1; TFDP2; PMS2; ALK; FAS; TP53; RICTOR; CCNB1; PPP2R1A; ATR; TTC5; CNOT6; BAX; SUPT16H; SMYD2; PDPK1; CCNT1; RBBP4; SSRP1; PMAIP1; MLH1; CNOT1; ZNF385A; PPP2R5C; G6PD; PRR5; TAF12; RRAGA; BBC3; CDC25C; PRKAB2; GSR; SESN2; RAD9B; TAF4; COX6B1; PRDX5; TOPBP1; GTF2H3; POLR2D; TNRC6B; RHEB; COX11; YWHAZ; EXO1	Reactome
Cytosolic tRNA aminoacylation	0.00762633	0.836411609	0.525207006	1.7343984	384	24	AIMP2; QARS; YARS; AARS; EEF1E1; RARS; GARS; EPRS; PPA1	Reactome
Interferon alpha/beta signaling	0.006126872	0.836411609	0.404884881	1.631392666	310	56	IFNAR2; GBP2; RNASEL; OAS2; OAS3; SOCS3; STAT2; IFIT1; PSMB8; HLA-C; PTPN6; IFITM3; IFITM1; HLA-B; EGR1; IRF1; HLA-A	Reactome
Steroid hormones	0.00383331	0.836411609	0.485293294	1.749989429	193	34	HSD11B2; STAR; STARD3NL; CYP11A1; CYP21A2; HSD17B2; HSD11B1; CUBN; FDXR	Reactome
Apoptosis	0.002525053	0.431784108	0.380687215	1.649678682	127	82	CASP4; TRAF3; MAPK10; BID; CYCS; TNFRSF1A; CASP6; TNFRSF25; TP73; TNFRSF10B; BAD; MYC; IKBKG; CASP1; NFKBIE; IRF1; DFFB; BCL2L1; FAS; TP53; TRAF1; BAX; TRADD; PMAIP1; NFKBIB; HELLS; BCL2L2; BBC3; TRAF2; PIK3R1; NFKBIA	Wikipathways
Integrated Breast Cancer Pathway	0.006332746	0.721933071	0.39496863	1.6158355	319	61	AHR; KRAS; CDH1; EP300; FOSL1; ODC1; MYC; RAD54L; RASGRP3; XRCC3; GADD45A; CREB1; NUP85; ANXA1; GDI1; IMPA1; AURKA; RAP1A; BAX; TFPI; GRN	Wikipathways
TP53 Regulates Transcription of Cell Death Genes	0.001051295	0.359542984	0.558943936	1.919600523	52	28	BID; CASP6; TNFRSF10B; TNFRSF10A; CASP1; TP53I3; TNFRSF10D; IGFBP3; FAS; BAX; PMAIP1; BBC3; BCL6; STEAP3; TP53AIP1	Wikipathways

Supplementary Table 3: HNSC gene set over-representation (p <= 0.01). P values are calculated by the one-sided Fisher's exact test with the hypergeometric distribution. P value (adjusted) are adjusted for multiple comparisons.

**Pathway**	**Gene set size**	**p value**	**p value (adjusted)**	**Jaccard Index**	**Overlapping**	**Number of overlapping**	**Genes**	**Source**
Beta oxidation of butanoyl-CoA to acetyl-CoA	5	0.00370472	1	0.001745201	ECHS1; ACSM3; HADH	3	lowriskgenes	REACTOME
Regulation of TP53 Activity through Acetylation	30	0.004941329	1	0.004602992	HDAC1; AKT1; MAP2K6; EP300; PIP4K2B; ING5; BRD1; ING2	8	lowriskgenes	REACTOME
PI5P Regulates TP53 Acetylation	9	0.005029165	1	0.00232288	MAP2K6; EP300; ING2; PIP4K2B	4	lowriskgenes	REACTOME
DNA Mismatch Repair	9	0.008339762	1	0.002321532	PCNA; MSH6; RFC1; POLD1	4	lowriskgenes	Wikipathways
Hematopoietic Stem Cell Gene Regulation by GABP alpha-beta Complex	19	0.008593	1	0.003466205	DNMT3B; GZMB; SMAD4; GABPB1; EP300; CREBBP	6	lowriskgenes	Wikipathways
Sulfur relay system - Homo sapiens (human)	8	0.005018461	0.818003735	0.00232423	MOCS3; MPST; CTU1; TST	4	highriskgenes	KEGG
African trypanosomiasis - Homo sapiens (human)	34	0.005096596	0.818003735	0.005166475	IDO1; APOA1; PRKCA; IFNG; HPR; THOP1; HBA1; HBA2; IL6	9	highriskgenes	KEGG
Synthesis of PE	14	0.004319961	1	0.002900232	CPT1B; PHOSPHO1; PCYT2; ETNK2; ETNK1	5	highriskgenes	REACTOME
Scavenging of heme from plasma	12	0.004319961	1	0.002900232	LRP1; HPR; HBA1; HBA2; APOA1	5	highriskgenes	REACTOME
RUNX1 and FOXP3 control the development of regulatory T lymphocytes (Tregs)	10	0.008322419	1	0.00232288	CTLA4; IL2RA; IFNG; CBFB	4	highriskgenes	REACTOME
G2 Phase	5	0.008557166	1	0.001745201	CCNA2; CDK2; E2F1	3	highriskgenes	REACTOME
Reelin signalling pathway	5	0.008557166	1	0.001745201	DAB1; RELN; VLDLR	3	highriskgenes	REACTOME
Glycerophospholipid biosynthesis	133	0.010844308	1	0.01103144	HADHA; GPAM; LPCAT4; ETNK2; PCYT1A; ETNK1; PCYT2; CSNK2A2; CPT1B; PITPNB; PTDSS1; LCLAT1; PITPNM3; CPNE1; DDHD2; LPCAT3; PHOSPHO1; SLC44A5; PEMT; CPNE3	20	highriskgenes	REACTOME
Kennedy pathway from Sphingolipids	13	0.000916315	0.550705476	0.003480278	PCYT1A; PTDSS1; PEMT; PCYT2; ETNK2; ETNK1	6	highriskgenes	Wikipathways
Interleukin-10 signaling	38	0.007625464	1	0.00516055	CCR1; CCL20; CCL22; CXCL1; CXCL2; TNFRSF1A; CSF1; CSF3; IL6	9	highriskgenes	Wikipathways
Tumor suppressor activity of SMARCB1	31	0.007744666	1	0.004600345	SMARCC1; EED; H3F3A; H3F3B; GLI4; CDK6; CDK4; ACTL6A	8	highriskgenes	Wikipathways
Cytokines and Inflammatory Response	28	0.008568904	1	0.003468208	CSF1; IFNG; CXCL1; CXCL2; CSF3; IL6	6	highriskgenes	Wikipathways
Signaling by PTK6	2	0.010001747	1	0.001164822	SOCS3; PTK6	2	highriskgenes	Wikipathways

Supplementary Table 4: HNSC gene set enrichment analysis (GSEA) (p <= 0.01). P values are calculated by the permutation test (nperm=100000). P value (adjusted) are adjusted for multiple comparisons.

Pathway	p value	p value (adjusted)	ES	NES	n More Extreme	Size	Leading Edge Genes	Source
B cell receptor signaling pathway - Homo sapiens (human)	0.009615951	0.622169841	-0.372038015	-1.563666442	489	69	PPP3CC; NFKBIA; RAF1; SYK; INPP5D; FCGR2B; CD79A; CD19; RASGRP3; AKT1; RAC2; PIK3R2; MAP2K2; MAPK1; CARD11; PIK3CB; BLNK; MALT1; SOS2; PIK3CA; PPP3CA	KEGG
Cell cycle - Homo sapiens (human)	0.00644264	0.622169841	0.325408317	1.516089026	314	118	CDK6; CCNA2; CCNB2; PTTG2; ESPL1; YWHAG; E2F1; GADD45A; MCM6; ANAPC7; YWHAB; CCNB3; TTK; CDK2; SKP1; CDC20; CDK4; CDKN2B; CHEK2; FZR1; CUL1; PRKDC; CDC7; E2F5; WEE1; CDKN1C; HDAC2; ANAPC10; CCNH; MDM2; YWHAZ; TP53	KEGG
Autophagy - animal - Homo sapiens (human)	0.009747426	0.622169841	-0.321369757	-1.479444734	498	115	PDPK1; ATG2B; GABARAPL2; RAF1; GABARAPL1; STK11; ATG9A; ATG7; ATG5; ATG16L1; AKT1; MAPK10; PIK3R2; RRAGD; MAP2K2; MAPK1; RRAGB; MAPK8; PIK3CB; SNAP29; ATG4A; RPS6KB1; MAP3K7; DDIT4; ATG2A; IRS1; PIK3CA; CAMKK2; GABARAP; RB1CC1; ITPR1; AKT3; PPP2CA; PPP2CB; TRAF6; NRBF2; PRKACA; NRAS; BAD; MTOR	KEGG
African trypanosomiasis - Homo sapiens (human)	0.006396588	0.622169841	0.472828815	1.711557555	314	34	PRKCA; HBA1; THOP1; HBA2; APOA1; IDO1; IFNG; HPR; IL6; IL18; ICAM1; IL12B; GNAQ; FAS; APOL1; PRKCG; IL1B; SELE; PLCB3; VCAM1; TNF; PLCB2; HBB	KEGG
Neutrophil degranulation	0.001029866	0.489701339	0.255683343	1.407114861	48	448	CD58; PGAM1; SNAP23; S100A9; XRCC5; ALDH3B1; DYNLT1; MMP8; FUCA2; MME; PRG2; TRAPPC1; HSPA8; DBNL; PRDX6; PSMD11; FTL; TMEM63A; METTL7A; RNASE3; ATP11B; TRIM24; TMEM179B; ATP6V0C; ATP8B4; SVIP; IGF2R; ORM1; CPNE1; PSMA5; DSG1; TLR2; GLA; IQGAP1; DYNC1H1; DNAJC13; GYG1; DEFA1B; RAP1B; PSMD12; GLIPR1; CPNE3; PA2G4; MOSPD2; SLC11A1; GLB1; PGLYRP1; GCA; FAF2; CD44; C5AR1; PLAUR; SIRPB1; CTSB; CXCL1; ADAM8; TMEM173; KRT1; PSMD7; RAB9B; C1orf35; LAMP1; SELL; RAB5B; MAN2B1; FPR2; CD68; PYGB; NCSTN; A1BG; FCGR3B; PSMD2; PPBP; AGA; NME1-NME2; ANO6; TICAM2; CAB39; PNP; TCIRG1; PSMC3; ALOX5; CXCR1; CAPN1; DOK3; HSP90AA1; COTL1; MGST1; PTPRC; NFAM1; CYFIP1; APRT; DNAJC5; S100A8; ARL8A; QSOX1; TUBB; CR1; RHOF; OSCAR; PLD1; STK10; ATAD3B; RHOG; PPIE; PSMD13; RAB10; STBD1; PYGL; PSEN1; EEF2; CD93; SLCO4C1; MNDA; ALDOC; PTX3; CTSC	Reactome
Synthesis of Leukotrienes (LT) and Eoxins (EX)	0.009007588	0.939960889	-0.555523767	-1.724026397	456	19	ALOX15; CYP4F3; GGT5; CYP4B1; ALOX5AP; DPEP1; GGT1; MAPKAPK2; CYP4F22; DPEP3	Reactome
NOTCH3 Intracellular Domain Regulates Transcription	0.007386049	0.939960889	-0.554602994	-1.74419649	373	20	PBX1; MAMLD1; EP300; HEY1; CREBBP; MAML3; SNW1; NOTCH3; MAML2; KAT2A	Reactome
Common Pathway of Fibrin Clot Formation	0.005393678	0.939960889	0.599478446	1.811376587	265	17	SERPINC1; PROCR; PROS1; PF4V1; PROC; F2; F13A1	Reactome
Mitotic Prometaphase	0.005201801	0.939960889	0.300752591	1.471925804	252	165	NCAPG; TUBA1A; SPC24; NEK2; CCNB2; KIF2C; ZW10; CLIP1; YWHAG; PRKAR2B; PPP2R5E; CSNK1E; DYNC1H1; RANGAP1; CDCA8; ZWILCH; ODF2; CEP72; CENPQ; CEP152; CDC20; CSNK2A2; CKAP5; NSL1; AHCTF1; SDCCAG8; CEP135; SKA1; CEP192; CEP250; SMC2; SSNA1; NDE1; SPC25; ERCC6L; CSNK1D; CENPO; HSP90AA1; AURKB; TUBG2	Reactome
Intraflagellar transport	0.000511398	0.486339765	-0.517400034	-1.926267214	25	39	IFT81; TTC26; TNPO1; IFT74; DYNLL2; IFT46; IFT20; TTC21B; TRIP11; CLUAP1; IFT122; TCTEX1D2; TCTEX1D1; KIF3B; DYNLRB1; IFT172; WDR34; KIF3A	Reactome
Cell Cycle	0.004008222	0.685405961	0.337713106	1.563630653	194	114	CDK6; CCNA2; CCNB2; PTTG2; ESPL1; YWHAG; E2F1; GADD45A; MCM6; ANAPC7; YWHAB; CCNB3; TTK; CDK2; SKP1; CDC20; CDK4; CDKN2B; CHEK2; FZR1; CUL1; PRKDC; CDC7; E2F5; WEE1; CDKN1C; HDAC2; ANAPC10; CCNH; MDM2; YWHAZ; TP53	Wikipathways
Fatty Acid Biosynthesis	0.00303078	0.685405961	-0.569256695	-1.838913595	153	22	HADH; ECHDC2; ECH1; SCD; ECHS1; ECHDC3; ACAA2; ACLY; MECR	Wikipathways

Supplementary Table 5: Lung cancer gene set over-representation (p <= 0.01). P values are calculated by the one-sided Fisher's exact test with the hypergeometric distribution. P value (adjusted) are adjusted for multiple comparisons.

**Pathway**	**Gene set size**	**p value**	**p value (adjusted)**	**Jaccard Index**	**Overlapping**	**Number of overlapping**	**Genes**	**Source**
Oxygen-dependent asparagine hydroxylation of Hypoxia-inducible Factor Alpha	3	0.000999475	1	0.001747234	HIF1AN; HIF1A; EPAS1	3	lowriskgenes	REACTOME
PTK6 Expression	5	0.008557166	1	0.001745201	EPAS1; HIF1A; PELP1	3	lowriskgenes	REACTOME
GABA A (rho) receptor activation	3	0.010001747	1	0.001164822	GABRR1; GABRR2	2	lowriskgenes	REACTOME
Exercise-induced Circadian Regulation	48	0.005708783	1	0.006274957	STBD1; CBX3; PSMA4; CLDN5; KLF9; G0S2; HERPUD1; UCP3; ETV6; GSTM3; GFRA1	11	lowriskgenes	Wikipathways
Bladder cancer - Homo sapiens (human)	41	0.004980754	0.582276332	0.005730659	EGFR; MAP2K1; RB1; THBS1; MAPK1; HRAS; MDM2; FGFR3; E2F2; E2F3	10	highriskgenes	KEGG
One carbon pool by folate - Homo sapiens (human)	20	0.006359388	0.582276332	0.003474233	ALDH1L2; MTHFD1L; AMT; FTCD; GART; DHFR	6	highriskgenes	KEGG
Central carbon metabolism in cancer - Homo sapiens (human)	65	0.006432965	0.582276332	0.007373795	MAP2K1; HRAS; AKT2; PFKP; PFKM; HK3; LDHA; EGFR; FGFR3; G6PD; PDK1; MET; MAPK1	13	highriskgenes	KEGG
Tuberculosis - Homo sapiens (human)	178	0.008120665	0.582276332	0.014069264	AKT2; MRC1; IRAK1; RAB5C; CAMK2A; CAMK2B; TLR2; TLR1; VDR; CD209; CARD9; TNFRSF1A; STAT1; MAPK1; IL18; FCGR3A; FCGR3B; BAD; CEBPB; CEBPG; BID; NOD2; MALT1; NOS2; FCGR2B; CASP3	26	highriskgenes	KEGG
Pentose phosphate pathway - Homo sapiens (human)	30	0.009382451	0.582276332	0.004039238	H6PD; RBKS; DERA; TKTL1; PFKM; PFKP; G6PD	7	highriskgenes	KEGG
MicroRNAs in cancer - Homo sapiens (human)	299	0.01088367	0.582276332	0.013057671	HDAC4; DDIT4; MDM2; FGFR3; THBS1; CD44; PRKCG; HRAS; HMOX1; MAP2K1; SPRY2; FZD3; MAPK1; IGF2BP1; SLC7A1; CASP3; DICER1; MET; EGFR; PLCG2; PLAU; E2F3; E2F2; CCNE2	24	highriskgenes	KEGG
Signaling by cytosolic FGFR1 fusion mutants	18	0.004625502	1	0.003476246	ZMYM2; STAT1; TRIM24; GAB2; STAT5A; FGFR1OP	6	highriskgenes	REACTOME
Phase 0 - rapid depolarisation	44	0.006216062	1	0.005169443	SCN7A; CACNB4; SCN2A; FGF12; CAMK2A; CAMK2B; CACNA2D3; CACNA2D4; SCN5A	9	highriskgenes	REACTOME
Signaling by FGFR in disease	47	0.009134022	1	0.005163511	HRAS; GAB2; STAT1; FGFR3; ZMYM2; TRIM24; FGFR1OP; FGF5; STAT5A	9	highriskgenes	REACTOME
ERBB2 Regulates Cell Motility	15	0.009168191	1	0.002900232	ERBB4; EGFR; EREG; BTC; MEMO1	5	highriskgenes	REACTOME
FGFR1 mutant receptor activation	31	0.009382451	1	0.004039238	GAB2; STAT1; ZMYM2; TRIM24; FGFR1OP; FGF5; STAT5A	7	highriskgenes	REACTOME
Resolution of D-loop Structures through Holliday Junction Intermediates	33	0.009382451	1	0.004039238	GEN1; XRCC2; DNA2; BARD1; BLM; RAD51; EME2	7	highriskgenes	REACTOME
Signaling of Hepatocyte Growth Factor Receptor	34	0.0050574	0.850185102	0.005172414	HRAS; RAP1B; PTK2B; MET; JUN; MAPK1; RASA1; PTPN11; MAP2K1	9	highriskgenes	Wikipathways
ErbB Signaling Pathway	92	0.005787334	0.850185102	0.009518477	AKT2; CAMK2A; CAMK2B; MDM2; HRAS; JUN; BTC; MAPK1; PLCG2; MAP2K1; BAD; STAT5A; EREG; EGFR; ERBB4; PRKCG; PDK1	17	highriskgenes	Wikipathways
Osteopontin Signaling	13	0.006415057	0.850185102	0.002901915	MAP2K1; MAPK1; SPP1; PLAU; MAP3K14	5	highriskgenes	Wikipathways
Retinoblastoma Gene in Cancer	89	0.010481201	0.850185102	0.008963585	BARD1; MDM2; RBBP4; CCNE2; CHEK1; FAF1; POLE; FANCG; POLD3; DHFR; MSH6; RRM1; CCNA2; RB1; E2F3; E2F2	16	highriskgenes	Wikipathways

Supplementary Table 6: Lung cancer gene set enrichment analysis (GSEA) (p <= 0.01). P values are calculated by the permutation test (nperm=100000). P value (adjusted) are adjusted for multiple comparisons.

Pathway	p value	p value (adjusted)	ES	NES	n More Extreme	Size	Leading Edge Genes	Source
Non-small cell lung cancer - Homo sapiens (human)	0.010898204	0.321497006	0.374045141	1.557379546	545	66	E2F2; RB1; HRAS; PLCG2; E2F3; AKT2; MAP2K1; EGFR; MAPK1; BAD; PRKCG; STAT5A; PDPK1; NRAS; ALK; PIK3R2; GADD45A; TP53; RXRB	KEGG
Chronic myeloid leukemia - Homo sapiens (human)	0.001316629	0.186477295	0.405078284	1.734856777	65	76	E2F2; PTPN11; RB1; GAB2; HRAS; E2F3; AKT2; MAP2K1; CTBP1; MAPK1; MDM2; BAD; STAT5A; NRAS; BCL2L1; RUNX1; PIK3R2; GADD45A; TP53	KEGG
Melanoma - Homo sapiens (human)	0.003160632	0.186477295	0.409587644	1.682858843	157	62	E2F2; FGF5; RB1; HRAS; E2F3; AKT2; MAP2K1; EGFR; MAPK1; MET; MDM2; BAD; NRAS; PDGFB; PDGFC; PIK3R2; GADD45A; TP53; PTEN; CDH1; PDGFA; CDK6; FGF19; PIK3R1	KEGG
Bladder cancer - Homo sapiens (human)	0.006262965	0.263939228	0.450013135	1.68471731	313	40	E2F2; RB1; THBS1; HRAS; E2F3; MAP2K1; EGFR; FGFR3; MAPK1; MDM2; NRAS; TYMP; MMP9; TP53; HBEGF; CDH1	KEGG
Type I diabetes mellitus - Homo sapiens (human)	0.00769014	0.283573902	-0.4521162	-1.669986296	381	38	FASLG; PRF1; PTPRN; GZMB; IL12B; HLA-A; HLA-C; HLA-F; HLA-DPA1; HLA-DOA; GAD1; HLA-DQA2; FAS; ICA1; CD28; HLA-DRB1; PTPRN2; CD80; LTA; TNF; HLA-DPB1	KEGG
Glioma - Homo sapiens (human)	0.00201031	0.186477295	0.404008132	1.703994205	100	70	E2F2; CAMK2B; RB1; HRAS; PLCG2; E2F3; AKT2; MAP2K1; EGFR; MAPK1; MDM2; CAMK2A; PRKCG; NRAS; PDGFB; PIK3R2; GADD45A; TP53; PTEN; CALML3; PDGFA; SHC2; CDK6; PIK3R1; CALML6	KEGG
Tuberculosis - Homo sapiens (human)	0.002909468	0.186477295	0.314787174	1.522957884	145	159	CEBPB; CD209; CAMK2B; TLR1; VDR; BID; IRAK1; FCGR3A; STAT1; FCGR3B; AKT2; CARD9; CEBPG; NOS2; FCGR2B; MALT1; RAB5C; CASP3; MAPK1; MRC1; IL18; CAMK2A; TLR2; BAD; TNFRSF1A; NOD2; CD14; RFX5; JAK2; IRAK4; IL1B; CYP27B1; TCIRG1; ATP6V1H; RAB7A; LBP; IL10; C3; PLK3	KEGG
Phospholipase D signaling pathway - Homo sapiens (human)	0.00558147	0.263939228	0.318209685	1.501945651	279	134	PTPN11; ADCY4; GRM3; AGPAT1; GAB2; PTK2B; HRAS; PLCG2; PIP5K1A; AKT2; MAP2K1; F2R; EGFR; MAPK1; CXCR1; RALA; PLCB2; AGPAT5; DGKA; NRAS; ADCY3; AVPR1A; DGKQ; LPAR4; PLA2G4D; ADCY7; CYTH4; AGPAT4; PDGFB; DNM1; RRAS; LPAR2; PDGFC; PIK3R2; CYTH2; RRAS2; PTGFR; DGKG; GRM8; AGPAT2; PDGFA; LPAR6; SHC2; PLA2G4A; DGKD; PIK3R1; PLCB1; RALB	KEGG
MicroRNAs in cancer - Homo sapiens (human)	0.009658188	0.316573927	0.301807035	1.445153351	485	147	E2F2; SPRY2; IGF2BP1; CCNE2; DDIT4; HDAC4; THBS1; HRAS; PLCG2; E2F3; MAP2K1; FZD3; EGFR; FGFR3; CASP3; HMOX1; MAPK1; MET; SLC7A1; DICER1; CD44; MDM2; PLAU; PRKCG; NRAS; SERPINB5; MARCKS; CDCA5; ZFPM2; MMP9; PDGFB; PIK3R2; TP53; PTEN; TNXB; BMF	KEGG
Pentose phosphate pathway - Homo sapiens (human)	0.000559016	0.164909759	0.596920902	1.993460357	27	25	PFKM; DERA; TKTL1; PFKP; RBKS; H6PD; G6PD; PGM1	KEGG
FRS-mediated FGFR3 signaling	0.003178665	0.494390184	0.642618712	1.868421535	158	15	PTPN11; FGF5; HRAS; FGFR3; NRAS	Reactome
DNA Damage/Telomere Stress Induced Senescence	0.010010569	0.595003191	0.500076038	1.691868331	501	26	CCNE2; EP400; CCNA2; RB1; RAD50; HIST1H1E; H1F0; HMGA1; HIST1H1D; TP53; CDK2; CCNA1; HIST1H1B; LMNB1	Reactome
Mitotic G1-G1/S phases	0.000398153	0.378643096	0.387808644	1.739909886	19	98	E2F2; RBBP4; MCM5; CCNE2; FBXO5; CCNA2; RB1; DHFR; E2F3; POLE; AKT2; MYBL2; E2F5; LIN9; CDKN2C; PRIM1; MNAT1; CCNB1; PPP2R3B; POLE4; LYN; JAK2; SKP2; PRIM2; CDK2; POLA2; CDK6; PPP2R2A; RRM2; UBC; UBB; RPA2; MCM10; POLA1; CCNA1; POLE3	Reactome
DNA Replication	0.005616406	0.494390184	0.377161398	1.600913733	280	72	MCM5; CCNE2; CCNA2; DNA2; POLD3; POLE; ANAPC5; GINS2; GINS3; PRIM1; POLD1; POLE4; SKP2; PRIM2; CDK2; POLD4; RFC1; POLA2; UBE2C; ANAPC1; UBC; UBB; RPA2; MCM10; POLA1; CCNA1; POLE3; RFC4; LIG1; MCM4; CDC7; MCM2; CDC23	Reactome
Synthesis of DNA	0.005718498	0.494390184	0.384786885	1.611552406	286	67	MCM5; CCNE2; CCNA2; DNA2; POLD3; POLE; ANAPC5; GINS2; GINS3; PRIM1; POLD1; POLE4; SKP2; PRIM2; CDK2; POLD4; RFC1; POLA2; UBE2C; ANAPC1; UBC; UBB; RPA2; POLA1; CCNA1; POLE3; RFC4; LIG1; MCM4; MCM2; CDC23	Reactome
S Phase	0.000997865	0.474484603	0.377374196	1.680267365	49	94	MCM5; CCNE2; CCNA2; RB1; DNA2; POLD3; POLE; AKT2; ESCO2; ANAPC5; PDS5A; ESCO1; STAG1; GINS2; GINS3; PRIM1; MNAT1; POLD1; CDCA5; POLE4; SKP2; STAG2; PRIM2; CDK2; POLD4; RFC1; POLA2; PDS5B; UBE2C; ANAPC1; UBC; UBB; RPA2; POLA1; CCNA1; POLE3; RFC4; LIG1; MCM4; MCM2; CDC23; PTK6	Reactome
Signaling by EGFR	0.004887976	0.494390184	0.446018221	1.700639482	244	43	SPRY2; PTPN12; PTPN11; HRAS; EGFR; ADAM17; SH3GL2; NRAS; EPN1; ADAM10; PAG1; SH3KBP1; HGS; STAM2; PIK3R1; UBC; UBB	Reactome

Supplementary Table 6: Lung cancer gene set enrichment analysis (GSEA) (p <= 0.01). P values are calculated by the permutation test (nperm=100000). P value (adjusted) are adjusted for multiple comparisons. (continued)

Pathway	p value	p value (adjusted)	ES	NES	n More Extreme	Size	Leading Edge Genes	Source
G1/S Transition	0.008623104	0.546704814	0.370644315	1.564175082	430	70	MCM5; CCNE2; FBXO5; CCNA2; RB1; DHFR; POLE; AKT2; PRIM1; MNAT1; CCNB1; PPP2R3B; POLE4; SKP2; PRIM2; CDK2; POLA2; RRM2; UBC; UBB; RPA2; MCM10; POLA1; CCNA1; POLE3; MCM4; CDC7; MCM2; PTK6	Reactome
Signaling by SCF-KIT	0.002292435	0.494390184	0.468311909	1.776418915	114	42	CHEK1; PTPN11; SOCS6; GAB2; HRAS; SH2B3; STAT1; STAT5A; NRAS; VAV1; LYN; JAK2; MMP9; PIK3R2; GRAP; GRB7	Reactome
Signaling by FGFR in disease	0.002600676	0.494390184	0.479804168	1.768849487	129	37	FGF5; GAB2; HRAS; STAT1; TRIM24; FGFR1OP; FGFR3; ZMYM2; STAT5A; NRAS; ERLIN2	Reactome
FGFR1 mutant receptor activation	0.008454468	0.546704814	0.515043237	1.725252787	423	25	FGF5; GAB2; STAT1; TRIM24; FGFR1OP; ZMYM2; STAT5A; ERLIN2; FGFR1OP2; CPSF6; PIK3R1	Reactome
Signaling by FGFR1 in disease	0.003983826	0.494390184	0.494024464	1.76063992	199	32	FGF5; GAB2; HRAS; STAT1; TRIM24; FGFR1OP; ZMYM2; STAT5A; NRAS; ERLIN2; FGFR1OP2; CPSF6; PIK3R1	Reactome
Gastrin-CREB signalling pathway via PKC and MAPK	0.005151643	0.494390184	0.602598536	1.814066261	256	17	HRAS; EGFR; MAPK1; RPS6KA3; RPS6KA2; NRAS; RPS6KA1; HBEGF	Reactome
Resolution of D-loop Structures through Holliday Junction Intermediates	0.007078623	0.546704814	0.521156029	1.745728954	354	25	EME2; DNA2; BLM; BARD1; XRCC2; GEN1; RAD51; BRCA2; RAD50; XRCC3; TOP3A; PALB2	Reactome
Resolution of D-Loop Structures	0.008340317	0.546704814	0.50189357	1.714001031	417	27	EME2; DNA2; BLM; BARD1; XRCC2; GEN1; RAD51; BRCA2; RAD50; XRCC3; TOP3A; PALB2	Reactome
Fluoropyrimidine Activity	0.007188338	0.383959044	0.4843776	1.70963535	358	31	UPP2; DHFR; RRM1; GGH; UMPS; FPGS; XRCC3; TYMP; CES2; ABCC5; TP53; ABCG2; RRM2; ABCC3; TDG; UPP1	Wikipathways
Retinoblastoma Gene in Cancer	4.00E-05	0.013691501	0.431472262	1.891761227	1	86	FAF1; E2F2; RBBP4; CHEK1; FANCG; CCNE2; CCNA2; RB1; DHFR; POLD3; E2F3; BARD1; POLE; MSH6; RRM1; MDM2; PRIM1; CCNB1; ANLN; HMGB2; SKP2; TP53; DCK; ZNF655; CDK2; CDK6; MAPK13; RBBP7; RRM2	Wikipathways
Nanoparticle triggered autophagic cell death	0.003728145	0.318756355	-0.581397567	-1.832634063	186	20	TSC1; ATG7; ULK2; MAP1LC3A; ATG4A; TSC2; CHAF1A; SH3GLB1; BCL2	Wikipathways
Mammary gland development pathway - Pregnancy and lactation (Stage 3 of 4)	0.00056065	0.06391414	0.556819695	1.965323405	27	31	CEBPB; GAL; EGFR; ELF5; PNCK; ERBB4; ORAI1; BCL2L1; TNFSF11; JAK2; PRLR; ATP2C2	Wikipathways
Signaling of Hepatocyte Growth Factor Receptor	0.008262308	0.383959044	0.463582723	1.672953779	411	34	PTPN11; JUN; PTK2B; HRAS; RASA1; MAP2K1; RAP1B; MAPK1; MET	Wikipathways
Non-small cell lung cancer	0.008981498	0.383959044	0.37789964	1.576064839	449	66	E2F2; RB1; HRAS; PLCG2; E2F3; AKT2; PDK1; MAP2K1; EGFR; MAPK1; BAD; PRKCG; STAT5A; NRAS; ALK; PIK3R2; GADD45A; TP53; RXRB	Wikipathways
LTF danger signal response pathway	0.008870384	0.383959044	0.590021326	1.746165632	440	16	IRAK1; MAPK1; TLR2; CD14; IRAK4; IL1B; AGER; TREM1	Wikipathways

Supplementary Note 1

We compute the gradient of the meta-learning loss function. Suppose that, for each task T_τ, the inner-learner takes 2 steps of stochastic gradient descent and updates the parameters to θ_τ^2. From Equation (3), we can write the gradient using a Taylor expansion:

$$g = \frac{\theta_\tau^0 - \theta_\tau^2}{\alpha} = \mathcal{L}'_{\tau,0}\left(\theta_\tau^0\right) + \mathcal{L}'_{\tau,1}\left(\theta_\tau^0\right) - \alpha \mathcal{L}''_{\tau,1}\left(\theta_\tau^0\right)\mathcal{L}'_{\tau,0}\left(\theta_\tau^0\right) + O\left(\alpha^2\right)$$

$\mathcal{L}_{\tau,0}$ is the loss computed on the first minibatch sampled from task τ, and $\mathcal{L}_{\tau,1}$ is the loss computed on the second minibatch sampled from task τ. The expectation of the first two terms in Equation (5) corresponds to the gradient of expected loss, and the expectation of the third term can be written as:

$$\mathbb{E}_{\tau,0,1}\left[\mathcal{L}''_1(\theta)\mathcal{L}'_0(\theta)\right] = \frac{1}{2}\mathbb{E}_{\tau,0,1}\left[\frac{\partial}{\partial\theta}\left(\mathcal{L}'_1(\theta)\cdot\mathcal{L}'_0(\theta)\right)\right].$$

This term increases the inner product of the gradient of the first minibatch and the gradient of the second minibatch, which means it encourages the gradients from different minibatches for a given task to align in the same direction.
